# Developing a cure for Black Bone Disease

**DOI:** 10.1186/1750-1172-7-S2-A37

**Published:** 2012-11-22

**Authors:** Nicolas T Sireau

**Affiliations:** 1 Alkaptonuria Society, Cambridge, CB2 9JG, UK

## 

Alkaptonuria (AKU for short) was the first genetic disease ever identified as such, by Dr Archibald Garrod in 1901 in London [1]. It is a rare disease affecting approximately one in 250,000 people, apart from countries such as Slovakia, Jordan and parts of South India where the number is up to 10 times higher [2]. Alkaptonuria is a monogenic disease leading to an enzyme deficiency, causing the accumulation of homogentisic acid (HGA) at 2,000 times the normal rate [3]. The HGA binds to cartilage and bone and pigments, turning it black in a process called ochronosis - hence its name of Black Bone Disease. The AKU Society, a patient advocacy group, has worked in close partnership with the Royal Liverpool University Hospital and the University of Liverpool over the past nine years to develop a major programme of research and treatment. This started with the post-mortem of an AKU patient [4], funded through sponsored events, followed by the funding of a PhD programme that developed an *in vitro* model of AKU [5]. Thanks to support from the Big Lottery Fund, the AKU Society then funded a four-year programme at UoL that successfully created an animal model of AKU, in which new therapies are being tested. The AKU Society and RLUH in parallel launched a global campaign to identify AKU patients [6], starting with three patients in the UK and reaching more than 1,000 patients globally by 2012. AKU patients and their families set up formal AKU Societies in the UK, France, Germany, the Netherlands, Italy, the USA and Canada in order to build the patient movement. A study was carried out to find out the average cost of an AKU patient to the National Health Service: £100,000 a year. This was used to build a case to the NHS for funding the National Alkaptonuria Society at RLUH and launching it in June 2012. The AKU Society, RLUH and UoL led the creation of an international consortium including 15 pharma companies, biotechs, universities, clinical trial centres, patient groups and contract research organisations in eight countries across Europe and North America. Thanks to funding from the European Commission, this consortium will launch in late 2012 a five-and-a-half year clinical development programme to develop and obtain marketing authorisation for nitisinone, a small molecule that inhibits the accumulation of homogentisic acid. Further AKU research centres have also been established in Jordan and South India.
